# Ruptured and Crippled

**Published:** 1872-12

**Authors:** 


### RUPTURED AND CRIPPLED.

On the corner of Lexington Avenue and 42d Street, New York, is an immense and magnificent building, erected by the princely munificence of New Yorkers, for the purpose of affording to unfortunates the very best medical and surgical appliances in the world. Persons are charged according to their ability to pay; those too poor to pay are kindly received and cared for. The institution is under the care, control, and patronage of citizens of the highest standing socially and financially.

The great-hearted John C. Green is president; the following names are given as vice-presidents, every name representing one of nature's noblemen, in the highest sense of the term, to wit: James Lenox, George Griswold, John David Wolfe, Stewart Brown, and A. R. Wetmore. Come to think of it, can't exactly determine whether they deserve quite as much to be said of their liberality, because they couldn't help it: it was born in them; they inherited it from their fathers. If John C. Green and James Lenox were prevented from giving money away, a thousand dollars at a time, ten thousand, a hundred thousand at a time, as they have done, and will do again, they would be in the same category with that black scamp who killed his beautiful wife, had no occupation, and became the most miserable creature in existence. And only think of A. R. Wetmore being cut off from all opportunity of aiding and abetting, by his time and personal labor, and sympathies and money, every good work having for its object the help and elevation of the sick and suffering poor! Why, he would consider himself one of the most unfortunate of men! And just look at some of the names who have aided this great charity by their free-will offerings: William H. Bradford, Stewart Brown, William E. Dodge, Thomas H. Faile — an old bach of Fifth Avenue. Why in the world don't he get married to some noble-hearted Christian woman of position and culture, who would follow in the footsteps of that generous worker, Mrs. Marshall O. Roberts, who supervises the princely liberalities of her husband! Then there are Fisk and Hatch, the bankers, Presbyterian ministers' sons, by the way, and Edward S. Jaffray, another Presbyterian; how these large hearted sons of Presbyters launch out their dollars by the bushel! only present a worthy object. Frederick Manquard and C. V. S. Roosevelt, Paul Spofford and A. T. Stewart, James Suydam and Samuel Willetts, and Eli White, give a thousand dollars apiece; and the modest name of "A Friend" comes down for another thousand, as an investment in the bank of heaven, which always pays large dividends in perpetuity; H. K. Corning and John David Wolfe three thousand each; Mrs. Ruth Tarbell and Jonathan Sturges, with H. and C. Rose, Miss Mary J. Gelston, and J. C. Green, five thousand dollars each; and John C. Baldwin gives fifteen thousand, to secure a house and home and medical aid to the unfortunate of their race, whom they have never seen. May the blessings of the good follow them all, evermore!

